# Source-Sink Colonization as a Possible Strategy of Insects Living in Temporary Habitats

**DOI:** 10.1371/journal.pone.0127743

**Published:** 2015-06-05

**Authors:** Jan Frouz, Pavel Kindlmann

**Affiliations:** 1 Institute for Environmental Studies, Charles University, Prague, Czech Republic; 2 Institute of Soil Biology, Academy of Sciences of the Czech Republic, České Budějovice, Czech Republic; 3 Department of Biodiversity Research, Global Change Research Centre, Academy of Sciences of the Czech Republic, Brno, Czech Republic; Federal University of Viçosa, BRAZIL

## Abstract

Continuous colonization and re-colonization is critical for survival of insect species living in temporary habitats. When insect populations in temporary habitats are depleted, some species may escape extinction by surviving in permanent, but less suitable habitats, in which long-term population survival can be maintained only by immigration from other populations. Such situation has been repeatedly described in nature, but conditions when and how this occurs and how important this phenomenon is for insect metapopulation survival are still poorly known, mainly because it is difficult to study experimentally. Therefore, we used a simulation model to investigate, how environmental stochasticity, growth rate and the incidence of dispersal affect the positive effect of permanent but poor (“sink”) habitats on the likelihood of metapopulation persistence in a network of high quality but temporary (“source”) habitats. This model revealed that permanent habitats substantially increase the probability of metapopulation persistence of insect species with poor dispersal ability if the availability of temporary habitats is spatio-temporally synchronized. Addition of permanent habitats to a system sometimes enabled metapopulation persistence even in cases in which the metapopulation would otherwise go extinct, especially for species with high growth rates. For insect species with low growth rates the probability of a metapopulation persistence strongly depended on the proportions of “source” to “source” and “sink” to “source” dispersal rates.

## Introduction

Continuous colonization and re-colonization is critical for the survival of insect species living in temporary (“source”) habitats [1–3]. When populations in these temporary habitats are depleted, some insect species escape extinction by surviving in permanent, but less suitable (“sink”) habitats, where they achieve lower growth rates than in temporary habitats and sometimes long-term population survival can be maintained here only by immigration [4–9], It is well known that permanent sink habitats can significantly affect metapopulation dynamics of an insect species, as e.g. (i) their utilization can maximize the total number of offspring produced in a landscape [4, 5, 10] and (ii) dispersal to permanent habitats can reduce competition in temporary habitats and at the same time ensure survival of some additional offspring [2]. However, their importance in ensuring the survival of insect species that are temporary habitat dwellers, and especially under what conditions they are important is unknown mainly because they are difficult to study experimentally.

Many theoretical models assume that the depletion of habitats occurs at random [11–13]. If this is the case, then there are always some habitats that remain habitable, where the species in question can survive and eventually recolonize other suitable habitats. However, quite often the depletion of suitable “source” habitats is synchronized, e.g. due to seasonal changes in climatic conditions (periodical drought, frost or similar), and only a few permanent habitats remain suitable for the species. At the same time many species may occur not exclusively in one optimal habitat but also in set of suboptimal habitats. These habitats often differ in resistance to major disturbances and consequently some of them may be more permanent than the optimal habitat. It has been recorded for many Diptera, Lepidoptera and Orthoptera that such permanent suboptimal habitats play an important role in the species survival [14–20]. Nice example are terrestrial chironomids living in the surface layer of soil that periodically dry out [19]. In such cases, recolonisation of such habitats from another similar habitat is impossible, as all populations in these habitats were depleted and therefore can only be recolonized by emigrants from another type of habitat, where the population has survived. However, the relative importance of factors determining the success of recolonisation is unknown, again mainly because it is difficult to study them experimentally in the field.

Here we aim to overcome this problem by using a simulation model to investigate, how environmental stochasticity, species population growth rate and ability of the species to disperse over short or long distances affect the likelihood of population persistence in a network consisting of temporary and permanent habitats. We considered the consequences of both simultaneous and random occurrence of depletion of populations in temporary habitats.

## The Model

We assumed ω temporary habitats, *H*_1_, *H*_2_,…, *H*_ω_, each of which are associated with an adjacent permanent habitat (scenario A) or no permanent habitat (scenario B). We used ω = 12. The population dynamics within each temporary habitat, *H*_*i*_, is described as:

$$N_{sour,i,n+1}=\left(N_{sour,i,n}(1-E_{sour})+E_{\sin}\;.N_{\sin,i,n}+\sum_{j=-m}^{m}\frac{m+1-\left|j\right|}{m(m+1)}.\frac{2m}{2m+1}E_{sour}.N_{sour,i+j,n}-\frac{1}{n}E_{sour}.N_{sour,i,n}\right).S.e^{r_{sour}(1-N_{sour,i,n}/K))}$$ (1)

and that in the permanent habitat adjacent to a temporary habitat *H*_*i*_ as

$$N_{\sin,i,n+1}=\left(N_{\sin,i,n}(1-E_{\sin})+\frac{1}{2m+1}E_{sour}.N_{sour,i,n}\right).e^{r_{\sin}}\;,$$ (2)

where *N*_*sour*,*i*,*n*_ (*N*_*sin*,*i*,*n*_) are the numbers of individuals in the temporary (permanent) habitat *i* at time *n*, respectively, *E*_*sour*_ and *E*_*sin*_ are the rates of dispersal from temporary (source) and permanent (sink) habitats respectively, *S* is the probability of a population persisting in the temporary habitat at each step, *K* is the carrying capacity (set equal to 1000 in all habitats and simulations) and *r*_*sour*_ is the growth rate in the temporary habitat (set equal to 1 for "low" and 3 for "high" growth rate). The growth rate in the permanent habitat was set to *r*_*sin*_ *=* -0.5, to simulate the sub-optimal conditions in the permanent habitats, compared to temporary ones (i.e., *r*_*sin*_ *< r*_*sour*_). The growth rates were chosen arbitrarily, but within the range of realistic values. Their exact value depends on time unit anyway, so their choice cannot have an effect on qualitative outcomes of the simulations. See Table 1 for summary of the parameter values used.

**Table 1:** Parameter values used in the model.

Parameter	Symbol	Unit	Value used here
**Number of temporary habitats**	ω	number	12
**Carrying capacity of temporary habitats**	K	Individuals per habitat	1000
**Maximum distance individuals disperse**	m	Number of neighbouring habitats	1, 3
**Population growth rate in temporary habitats**	r_sour_		1, 3
**Population growth rate in permanent habitats**	r_sin_		-0.5
**Proportion of individuals dispersing from temporary habitats**	E_sour_	Proportion 0–1 scale	0 to 1 with step 0.2
**Proportion of individuals dispersing from permanent habitats**	E_sin_	Proportion 0–1 scale	0 to 1 with step 0.2
**Initial population size in temporary habitats**	N_sour,i,0_	Individuals	100
**Initial population size in permanent habitats**	N_sin,i,0_	Individuals	0

The biological meaning of Eqs (1) and (2) is that the animals disperse between temporary habitats in the vicinity of where they developed and if a permanent habitat(s) is present, also between temporary and adjacent permanent habitats. The number of animals *emigrating* from each temporary habitat is linearly dependent on *N*_*n*_ and on emigration rate (proportion of population that leave the habitat in which they developed in each generation), *E*_*sour*_. Number of animals arriving in each temporary habitat is calculated as

$$E_{\sin}.N_{\sin,i,n}+\sum_{j=-m}^{m}\frac{m+1-\left|j\right|}{m(m+1)}E_{sour}.\frac{2m}{2m+1}N_{sour,i+j,n}-\frac{1}{m}E_{sour}.N_{sour,i,n}\;,$$ (3)

which is the sum of the immigration rates from the permanent (if present), *E*_sin_.*N*_sin, *i*,*n*_, and temporary habitats, which is represented by the rest of Eq (3). The latter was chosen so, that the number of immigrants declined linearly with the distance between the temporary habitats and the maximum distance travelled was *m* habitats. This corresponds to a situation in which the habitats are assumed to be linearly arranged in one-dimensional space. The following boundary conditions were used: if *i*+*j* > *m*, then *i*+*j* was replaced by *i*+*j*-*k* and if *i*+*j* < 1, then *i*+*j* was replaced by *i*+*j*+*m*.

Number of animals emigrating from the temporary habitat to an adjacent permanent habitat (if present) was calculated as $\frac{1}{2m+1}N_{sour,i,n}$, so that the proportion $\frac{1}{2m+1}$ of animals emigrated to the permanent habitat and the remaining $\frac{2m}{2m+1}$ animals emigrated to the 2*m* surrounding temporary habitats.

We assumed two modes of dispersal: (i) short-range dispersal (*m* = 1), when organisms from a temporary habitat dispersed to the closest permanent habitat, at the same rate in both directions (i.e., half from *H*_i_ dispersed to *H*_i-1_ and the other half to *H*_i+1_), and (ii) long-range dispersal (*m* = 3), when the organisms from a temporary habitat dispersed to the closest permanent habitat, at the same rate in both directions, but the number decreased linearly with distance from the patch in which they developed, as described above. The dispersal from the permanent to neighboring temporary habitats was equal to *N*_sin,*i*,*n*_(1 − *E*_sin_), while that in the other direction by $\frac{1}{2m+1}N_{sour,i,n}(1-E_{sour})$, as follows from [1] and [2], i.e., the proportion emigrating to the permanent habitat was equal to the average proportion emigrating to neighboring temporary habitats.

It was assumed that only the temporary habitats were affected by “catastrophic disturbance events” (e.g. drought, flood or fire), which caused local extinction of the whole population in the affected patch. Catastrophes were assumed to occur at random with a probability that either remains constant in time and equal to 0.5 (scenario "random") or alternates between two values (we used 0.3 and 0.7) in consecutive generations (scenario "synchronized"). To simulate this, the value of *S* was always set equal to 0 or 1 at random with the given probability.

To summarize, for both A and B (permanent habitats present or absent), we had eight different scenarios: all combinations of "low" vs. "high" growth rates, "short-range" vs. "long-range" dispersers, and "random" and "synchronized" catastrophic events.

In theory, an increase in the probability of a metapopulation surviving, when permanent habitats are present, might be because there is a greater number of habitats in scenario A than in scenario B, rather than the *mixture* of temporary and permanent habitats in scenario A versus *only* temporary habitats in scenario B. To test, whether this is true, scenario A (permanent habitats present) was compared with scenario C, in which the permanent habitats were replaced by an equal number of temporary habitats, i.e., *n* temporary habitats, *H*_1_, *H*_2_,. ., *H*_ω_, each of which, *H*_*i*_, was accompanied by another *temporary* habitat, $H_{i}^{'}$. Dispersal was possible between habitats *H*_*i*_ and $H_{i}^{'}$, *H*_i-m_, *H*_i-m+1_,…, *H*_i+m_.

For each scenario, 200 simulations were performed. Probability of a metapopulation persisting was measured in terms of the proportion of the metapopulations surviving after 100 generations. Population in a given patch was recorded as extinct, when the number of individuals was less than 1. A metapopulation was recorded as extinct, if none of the populations in the temporary or permanent (if present) habitats survived. In each scenario, the difference in the proportion of metapopulations surviving in scenarios A (permanent habitats present) and B (permanent habitats absent) and between scenario C (satellite temporary habitats, $H_{i}^{'}$, present) and B (satellite permanent habitats, $H_{i}^{'}$, absent) was tested using χ^2^ tests.

## Results

**Fig 1:**
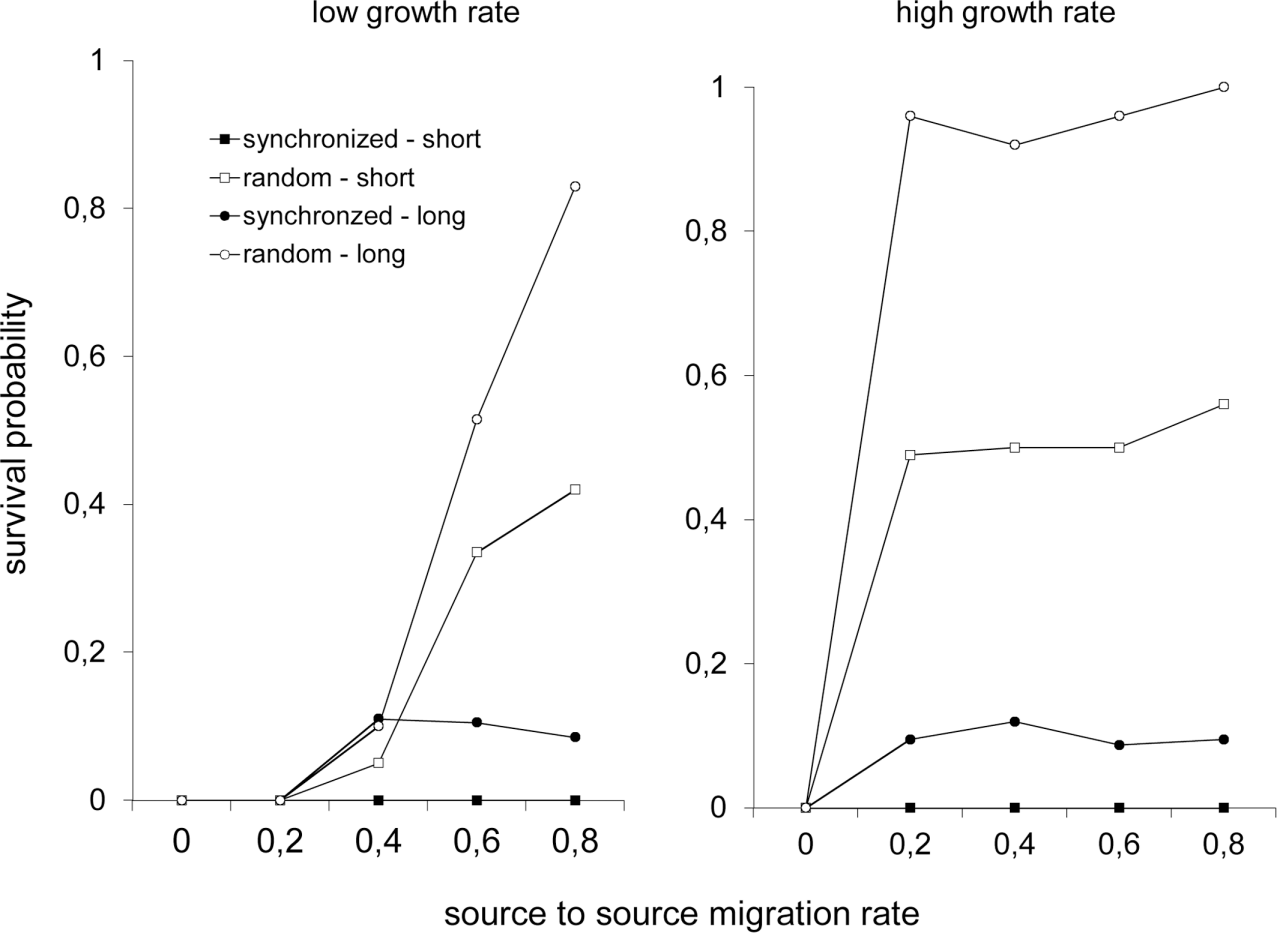
Predicted probability of a metapopulation surviving as a function of the rate of dispersal from habitats in a network consisting of only temporary habitats. Independently of the rate of dispersal, *E*, between habitats the probability of survival is lower, if environmental fluctuations are synchronized and individuals only disperse over short distances.

In the absence of permanent habitats (scenario B) metapopulations with either "low" or "high" population growth rates, consisting of long-range dispersers and in a randomly changing environment had the highest probability of persisting (Fig 1). Probability of persisting tended to increase with increasing rates of dispersal between habitats, *E*_*sour*_, and reached values close to 1 for large *E*_*sour*_ for long-range dispersers in a randomly changing environment. This was more pronounced for the higher growth rates (Fig 1). Probability of metapopulations consisting of short-range dispersers persisting in a random environment reached maximum values of only about 0.5. For a population living in an environment in which changes occurred synchronously the maximum probability of it persisting was much lower: about 0.1 when it consisted of long-range dispersers and 0 when it consisted of short-range dispersers.

**Fig 2:**
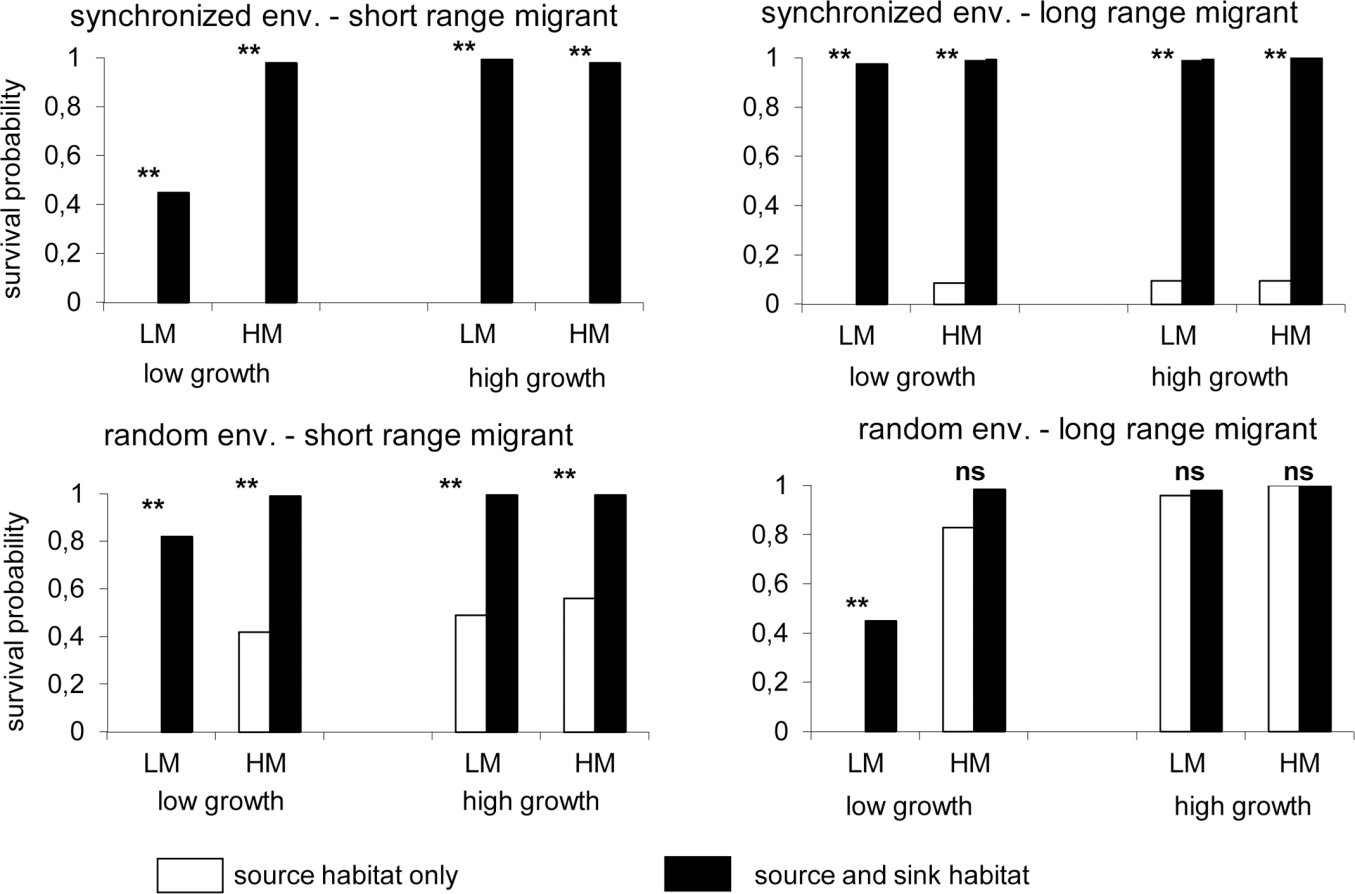
Comparison of the probability of survival of a metapopulation in a network without (white bars) and with a permanent habitat (black bars) for the different scenarios (see Methods); ** indicates significant differences (P<0.0001, χ^2^ test, d.f. = 199), ns means “non-significant”. Upper figures: synchronized environment; lower figures: non-synchronized environment. Figures on left: short-range dispersal; figures on right: long-range dispersal. Each diagram presents results for both low and high growth rates, and short (i.e. 0.2, LM) and long (i.e. 0.8, HM) range dispersal between habitats. Rate of immigration into the permanent habitat is 0.1 for HM and 0.7 for LM and from permanent to temporary habitats is 0.9 in all situations.

When permanent habitats were present (scenario A), the probability of a metapopulation in scenario B persisting was significantly greater for all the combinations that included either short range dispersers or a synchronized environment (χ^2^ test, d.f. 199, P <0.0001; Fig 2). Only in the combination long-range dispersers and a random environment was the effect of a permanent habitat mostly non-significant, except for those with a low growth rate and short-range dispersers (χ^2^ test, d.f. = 199, P <0.0001, see Fig 2). This effect was strongest for the combination with short-range dispersers living in a synchronized environment, when permanent habitats ensured a high probability of population persistence (close to 1) even in situations when a metapopulation was otherwise not able to persist (Fig 2).

**Fig 3:**
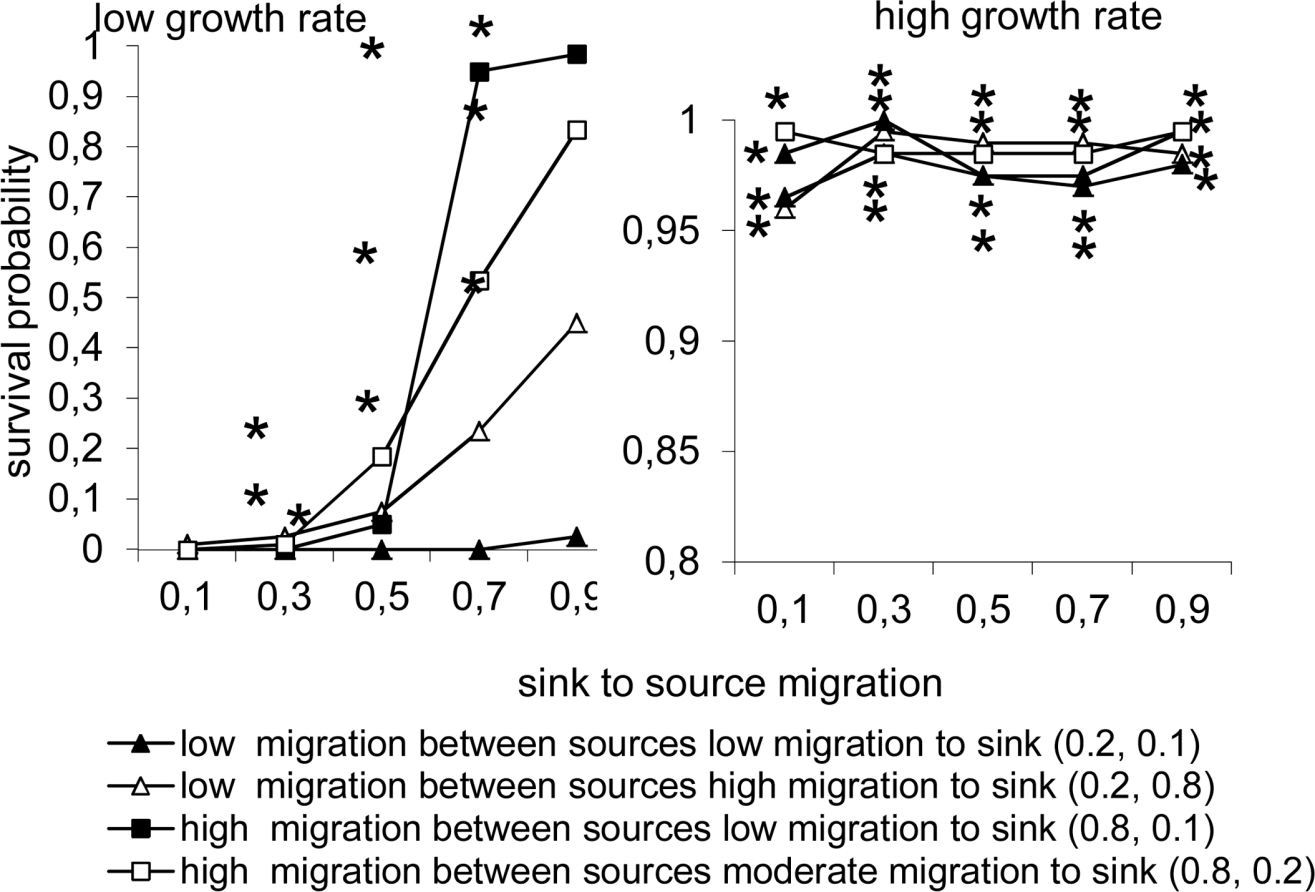
Predicted probability of a metapopulation surviving in which all the individuals only disperse over short distances in a network consisting of temporary and permanent habitats and in which the changes in the environment occur synchronously. Persistence is plotted as a function of both the dispersal and growth rates. The numbers in parentheses are the dispersal rates for the temporary to permanent and permanent to temporary habitats. * and ** indicate significant differences at P< 0.01 and P<0.001, respectively, for the above scenario and that without a permanent habitat, based on a χ^2^ test.

When the population growth rate was low the probability of a metapopulation persisting increased with increase in the dispersal rate, *E*_*sin*_, from permanent to temporary habitats (Fig 3). This increase was much less pronounced when the dispersal rate between permanent habitats was high (Fig 3). However, the effect of dispersal rate was insignificant when the population growth rate was high: in such cases the inclusion of a permanent habitat ensured high population persistence for all the combinations of dispersal rates considered.

**Fig 4:**
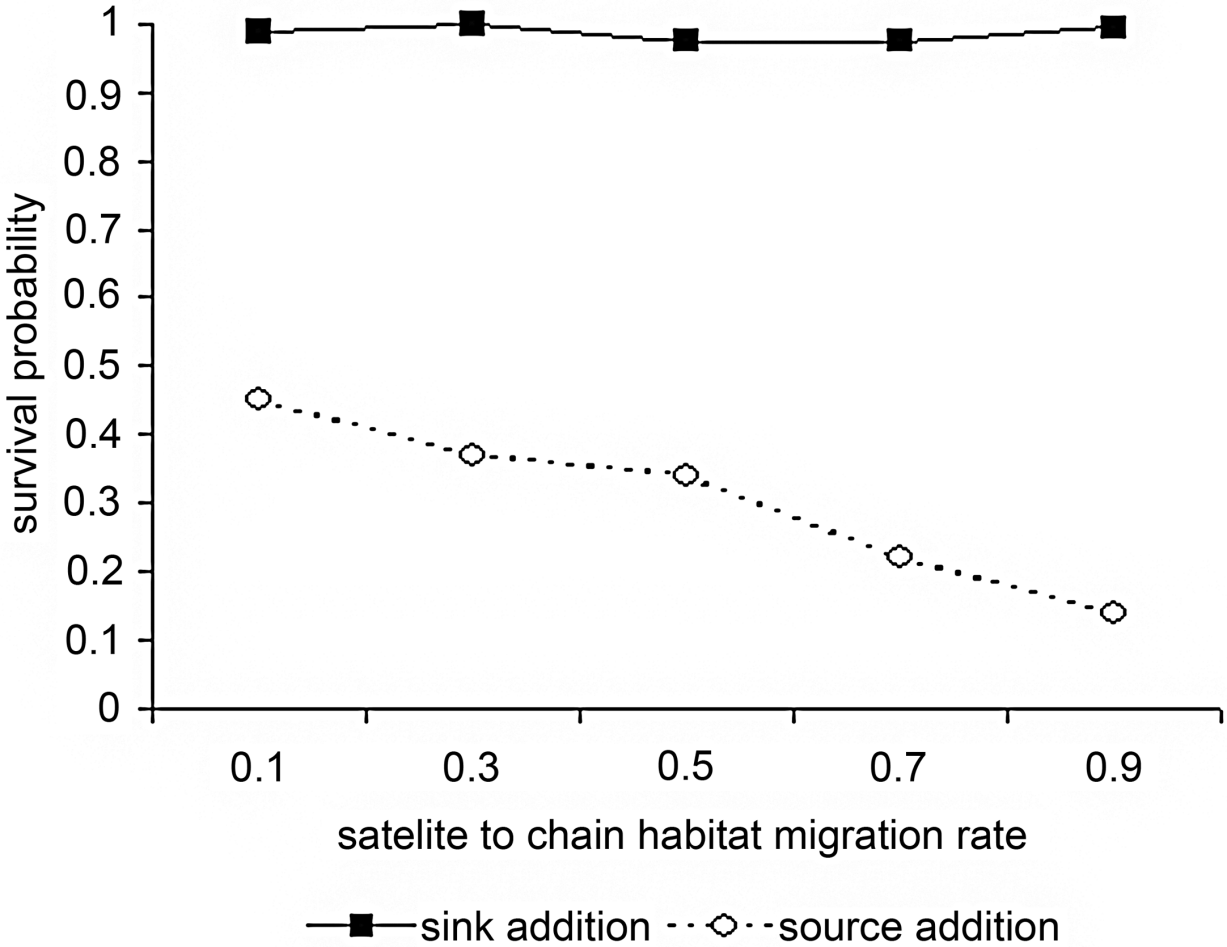
Predicted probability of a metapopulation surviving in which all the individuals only disperse over short distances in a network consisting of permanent and accompanying satellite habitats or temporary habitat in which the environment changes synchronously. Persistence is plotted as a function of both the dispersal and growth rates. Rate of dispersal along a chain of temporary habitats is 0.8 and a chain of temporary habitats with satellites 0.1.

Including satellite temporary habitats resulted in a significantly lower increase in the probability of survival than including satellite permanent habitats (χ^2^ test, d.f. = 199, P <0.0001 for all combinations of satellite to chain dispersal rate—Fig 4). Thus the stability of the permanent habitats was more important than the increase in the carrying capacity of the system or the number of system units per se.

## Discussion

Empirical data indicate that synchronized fluctuations in environmental conditions can reduce the likelihood of insect metapopulations persisting [1, 21, 22]. The effect of an extensive drought is a good example [15].

Here we demonstrate that when environmental disturbance simultaneously affects all the temporary habitats, then even an increase in the number of dispersers may not ensure population persistence. The insect species may, however, survive in some temporary habitats if they are not all equally affected by the environmental disturbance. Thus, diversity of habitats—presence of a mixture of good-quality temporary habitats and low-quality permanent habitats—is more important for metapopulation persistence than the same total number of good-quality temporary habitats.

The model presented indicates that growth rate and dispersal ability are positively correlated with the probability of metapopulation persistence, assuming everything else remains constant. When growth rate and/or dispersal ability is high, then the beneficial effect of the presence of permanent habitats on population persistence becomes insignificant. This is supported by empirical studies, which report source-to-sink recolonisation when dealing with populations living in habitats, where changes in environmental conditions are synchronized, usually because of some seasonal factor [14, 15, 18, 19, 23].

The exchange of dispersers between temporary and permanent habitats and vice versa is important for the success of the source-to-sink recolonisation strategy. In agreement with Griebel & Gottschalk [18], only a high rate of dispersal from permanent to temporary habitats can positively affect the persistence of a metapopulation. The required high rate of dispersal from permanent to temporary habitats does not conform to the definition of a sink habitat, for which immigration exceeds emigration [4, 5]. Nevertheless, the average population size in permanent habitats is low compared with that in temporary habitats due to the negative growth rate in the former. Thus, in absolute terms, the total number dispersing from source to sink habitats is greater than in the opposite direction, even if the rate of dispersal from sink to source is high.

Individuals generally tend to reproduce where conditions are optimal, regardless of where they develop. This assumption is supported by empirical data indicating that females that developed on optimal or sub-optimal foods prefer to oviposit where there is an optimal food supply [24]. This raises the question, why are some eggs laid in sub-optimal habitats? In some cases this may be due to competition [18]. However, oviposition on poor quality substrates is often recorded even when the females are offered a choice of an optimal or sub optimal substrate [25–27]. Moreover, the tendency to accept a low quality oviposition site increases if females are initially prevented from ovipositing [28, 29], or when their egg load is high [30, 31].

The model indicates that the presence of permanent habitats has a positive effect on the probability of metapopulation persisting, particularly when environmental conditions are synchronized or organisms only disperse over short-distances. However, when the population is not constrained by low dispersal ability and environmental conditions in the different patches are not synchronized, then including a permanent habitat did not result in an increase the probability of metapopulation persistence, which is consistent with other results [21, 22, 32, 33]. This cannot be accounted for in terms of an increase in the carrying capacity of the system, but rather in the stability of the permanent habitat.

Our study underlines the importance of sub-optimal habitats in the population biology of species with high population growth rates that occupy habitats frequently subjected to environmental disturbances. Spatio-temporal correlation of local populations is widely documented in insect population biology; therefore it can be expected that the mechanisms described in this study may have wide application in insect ecology including applied fields, such as pest and vector control or conservation biology.
